# Co-Metabolic Network Reveals the Metabolic Mechanism of Host–Microbiota Interplay in Colorectal Cancer

**DOI:** 10.3390/metabo16010064

**Published:** 2026-01-11

**Authors:** Han-Wen Wang, Wang Li, Qi-Jun Ma, Hong-Yu Zhang, Yuan Quan, Qiang Zhu

**Affiliations:** 1 Hubei Key Laboratory of Agricultural Bioinformatics, College of Informatics, Huazhong Agricultural University, Wuhan 430070, China; ygftfddd@webmail.hzau.edu.cn (H.-W.W.); wangli036@outlook.com (W.L.); mqj@webmail.hzau.edu.cn (Q.-J.M.); zhy630@mail.hzau.edu.cn (H.-Y.Z.); 2 Key Laboratory of Smart Farming for Agricultural Animals, College of Informatics, Huazhong Agricultural University, Wuhan 430070, China

**Keywords:** colorectal cancer, genome-scale metabolic model, co-metabolic network, host-microbiota interaction, key metabolites

## Abstract

Background: Colorectal cancer (CRC) is a malignancy that ranks among the top three in terms of both global mortality and incidence. Although numerous studies have demonstrated that gut microbes are implicated in CRC pathogenesis, the precise mechanisms underlying host–microbiota metabolic crosstalk remain poorly understood. Objective: This study aims to identify and delineate key co-metabolites and their associated metabolic pathways that modulate the biomass of CRC-related gut bacteria within healthy individuals, through the construction of host–gut microbiota co-metabolic network models. We seek to elucidate the underlying mechanisms of metabolic interplay between the host and CRC-related gut microbiota, thereby offering novel perspectives on the microbial involvement in the initiation and progression of CRC. Methods: We coupled a colon tissue-specific host Genome-Scale Metabolic Model (GEM), which utilized transcriptomic data from healthy human colon tissues, with 12 CRC-associated pro-/anti-carcinogenic gut bacterial GEMs to construct a co-metabolic network. Through a comparative analysis of the network structure and systemic methods (including Flux Sampling and metabolic difference analysis), we simulated scenarios of constrained host co-metabolite supply. Finally, metabolic subsystem enrichment analysis was employed to elucidate the specific molecular mechanisms by which key co-metabolites affect microbial function. Results: The 17 key co-metabolites identified include chloride ions, zinc ions, and acetate. Among these, thirteen metabolites (e.g., ferric iron, succinate, and acetate) were confirmed by literature to be associated with CRC. All 17 key co-metabolites were found to significantly modulate the biomass of CRC-associated gut bacteria. These regulatory effects primarily influence microbial function through core pathways such as glycerophospholipid metabolism and folate metabolism. Conclusion: This research provides a systemic perspective for elucidating the mechanisms of host–gut microbiota metabolic interplay in CRC, thereby complementing the existing theoretical framework concerning microbial regulation by the host genetic background.

## 1. Introduction

As the third most common malignancy and the second leading cause of cancer-related deaths worldwide, CRC arises from a complex interplay between the host genetic background and environmental factors [1,2]. While somatic mutations and epigenetic alterations serve as intrinsic drivers of tumorigenesis, the gut microbiota has been established as a pivotal extrinsic regulator [3]. Metagenomic studies have elucidated CRC-specific dysbiotic signatures, characterized by a significant depletion of beneficial short-chain fatty acid (SCFA)-producing commensals (e.g., Bifidobacterium) and an aberrant enrichment of pro-inflammatory, carcinogenic pathogens such as *Fusobacterium nucleatum* and *Bacteroides fragilis* [4,5]. Notably, this remodeling is not a random ecological drift but a directional selection process strictly governed by host genetic factors. Specifically, the host genotype imposes selection pressures on the microbiota by shaping specific intestinal biochemical environments and nutritional niches [6,7].

From a mechanistic perspective, host–microbiota interactions are fundamentally characterized by metabolic exchange across the shared interface of the intestinal lumen [8]. In this context, “co-metabolites” defined as small molecules that are exchanged, competed for, or synergistically utilized between the host and microbes—constitute a pivotal molecular link connecting host physiological states to microbial phenotypes [9]. By regulating the activity of metabolic enzymes or transmembrane transporters, the host directly modulates the bioavailability of specific nutrient substrates or signaling molecules within the lumen, thereby shaping the intestinal metabolic microenvironment [10,11]. These alterations impose specific selection pressures on the microbial community: the depletion or excessive accumulation of key co-metabolites can directly modulate the growth kinetics of specific strains, thereby restricting the colonization of beneficial bacteria or providing a competitive metabolic advantage to CRC-associated pathogens [12,13]. Consequently, systematically identifying key co-metabolites and quantitatively dissecting their regulatory effects on the growth kinetics of CRC-associated bacteria represent a critical yet unmet need in decoding the mechanisms of host-microbe metabolic interplay.

However, systematically quantifying and dissecting this interaction mechanism remains a formidable methodological challenge. Current research predominantly relies on statistical association studies; while capable of identifying correlations, these approaches struggle to establish underlying causal mechanisms [14,15]. In the field of computational biology, existing microbial metabolic models often oversimplify the host’s role, treating it merely as a passive nutrient absorber or a constant environmental boundary. This approach neglects the active remodeling of the gut microenvironment by host physiological metabolism, such as specific transport and enzymatic activities [16,17]. Consequently, there is a critical lack of mechanistic models capable of integrating multi-level “Host–Metabolite–Microbe” interactions, hindering the accurate simulation and prediction of how host-driven fluctuations in metabolic supply dynamically orchestrate CRC-associated microbiota succession.

To address these gaps, we developed a robust host–microbiome co-metabolic network by integrating a colon-specific GEM, constrained by healthy human colonic transcriptomics, with GEMs of 12 key CRC-associated (procarcinogenic or anticarcinogenic) bacteria. Using systems biology approaches, including flux sampling and differential metabolic analysis—we simulated restricted host co-metabolite supply scenarios to quantitatively dissect key metabolic interactions. This study provides a systems-level view of host–microbe metabolic interplay in CRC, offering novel insights into how host genetics mediate microbial regulation via metabolites and laying a theoretical foundation for metabolite-based precision prevention and therapy.

## 2. Materials and Methods

### 2.1. Model Data Collection and Preprocessing

#### 2.1.1. Human-GEM

For the host model, we employed the Human-GEM v1.18. This reconstruction encompasses 2889 genes, 4156 metabolites, and 12,995 metabolic reactions. To facilitate sophisticated intracellular metabolic simulations, metabolites within the model are precisely localized across multiple cellular compartments, including the extracellular space, peroxisome, mitochondrion, cytosol, lysosome, endoplasmic reticulum, Golgi apparatus, nucleus, and mitochondrial intermembrane space [18]. The integrity and consistency of the model were assessed using the MEMOTE standard quality control tool. All model files and associated parameters have been deposited in the project’s GitHub (v1.2.3) repository [19].

#### 2.1.2. Colonic Tissue Transcriptome Data

To accurately characterize the metabolic profile of the colon, we retrieved gene expression data from 406 healthy human colon tissue samples via the GTEx database [20]. Since a single gene often generates multiple transcripts and its cumulative expression is typically quantified by aggregating the expression levels of all associated isoforms, we calculated the mean Transcripts Per Million (TPM) value for each gene across all 406 samples to quantify cumulative expression. This approach ensured robust input data for constructing the colon-tissue-specific GEM, thereby accurately representing the stable gene expression profile within healthy colon tissue. Following the official Human-GEM guidelines, we employed the ftINIT algorithm to contextualize the model. Consequently, we employed a threshold of TPM > 1 for gene filtering, a strategy widely adopted in the construction of tissue-specific GEMs [21].

#### 2.1.3. GEMs of CRC-Associated Gut Microbiota

Given the scarcity of databases dedicated to detailing causal CRC–microbiota mechanisms, we systematically curated association data from MicroPhenoDB [22], gutMDisorder [23], and Peryton [24] (Supplementary Figure S1). To transcend the limitations of purely statistical associations, we performed a rigorous literature review to select strains based on three stringent criteria: (1) elucidated molecular mechanisms (involving metabolites, signaling pathways, or immune modulation); (2) definite pro- or anti-carcinogenic phenotypes; and (3) independent multi-level validation (spanning in vitro, in vivo, or epidemiological studies). Twelve core CRC-associated bacterial strains were ultimately identified (Table 1). Subsequently, the GEMs corresponding to these selected strains were retrieved from the AGORA2 repository to construct the host–gut microbiota co-metabolic model [25].

### 2.2. Construction of Host–Gut Microbiota Co-Metabolic Model

#### 2.2.1. Tissue-Specific GEM

To simulate host-microbe interactions, we constructed a colon tissue-specific host GEM based on Human-GEM v1.18 and colon gene expression data obtained from (GTEx Dataset) using the ftINIT algorithm. This algorithm optimizes the reconstruction by assigning weights to reactions based on gene expression levels to extract a core metabolic set [38,39]. Subsequently, we applied the E-flux algorithm to constrain metabolic fluxes. By mapping gene expression intensity directly to reaction flux boundaries, E-flux ensures the model accurately captures the metabolic transport profile specific to the colon tissue [40].

To validate the physiological relevance and effectiveness of the tissue-specific constraints, the resulting ColonSpecialGEM was compared with the generic Human-GEM v1.18. The comparison encompassed (1) structural metrics (e.g., number of genes, metabolic reactions, and the proportion of reactions with gene–protein–reaction associations); (2) functional capabilities (assessed by Flux Variability Analysis (FVA) to quantify the solution space); and (3) reaction essentiality, evaluated via single-reaction deletion analysis to identify critical reactions required for cellular maintenance. Differences in flux variability between the two models were evaluated for statistical significance using the Wilcoxon rank-sum test.

#### 2.2.2. Co-Metabolic Network Model

Next, a multi-species co-metabolic modeling approach based on the COBRA Toolbox was used to integrate the host tissue-specific GEM with the 12 CRC-associated bacterial GEMs, resulting in the host–gut microbiota co-metabolic network model. This method references the metabolic interaction theoretical framework proposed by Klitgord and Segrè, and incorporates the optimization strategy for multi-species co-metabolic models by Heinken et al., making it suitable for modeling and analyzing metabolic flux across multiple species [41].

The core function createMultipleSpeciesModel forms the foundation of this method. This function accepts the set of GEMs to be integrated, the list of biomass reaction names for each GEM, and the optional host model as input. It then calls multiple sub-functions, including createInterSpace (for building inter-compartment transport reactions), mergeTwoModels (for alignment and sparse matrix merging), and removeFutileCycles (for cycle pruning). The complete co-metabolic model (ModelJoint) can be mathematically represented as follows:

(1) $$M_{\mathrm{joint}}=F(\{M_{i}{\}}_{i=1}^{n},\{B_{i}{\}}_{j=1}^{n},H,N,K,R),$$

Here, $\{M_{i}{\}}_{i=1\;}^{n}$ represents the set of individual gut bacterial GEMs, $\{B_{i}{\}}_{j=1\;}^{n}$ represents the corresponding set of biomass reactions, H denotes the host model, N denotes the naming tags for the gut bacteria or host, K indicates whether gene information is merged, and R indicates whether futile cycles are removed.

During the integration process, a hierarchical metabolic space architecture was employed to describe the metabolic flow and exchange between the host and gut bacteria (Supplementary Figure S2). This architecture defines three metabolic compartments: the shared luminal space ([u]) to support metabolite exchange between the host and gut bacteria, the extracellular space ([e]) as the metabolic boundary for the host and each bacterium, and the host body fluid space ([b]) representing the host’s internal metabolite circulation. Each sub-GEM first extends its metabolic space via the createInterSpace sub-function, generating an extracellular space ([e]) connected to the shared luminal space ([u]). For the extracellular metabolites (met[e]) of each sub-GEM, corresponding shared luminal space metabolites (met[u]) are generated, and the stoichiometric matrix for transport reactions between the extracellular and luminal compartments is formulated as follows:

(2) $$S_{\mathrm{IEX}}=\left[\begin{array}{c} -I_{n\times n}\\ I_{n\times n} \end{array}\right],$$

where n is the total number of extracellular metabolites (met[e]) in the model, $I_{n\times n}$ identity matrix (ones on the diagonal, zeros elsewhere), $-I_{n\times n}$ denotes the flux of metabolites exiting the [e] compartment, and $I_{n\times n}$ represents the flux of metabolites entering the [u] compartment. This met[e]⇌met[u] transport reaction framework effectively simulates the metabolic exchange process between the host and gut bacteria. In this study, the construction of both tissue-specific GEMs and the co-metabolic models was performed within the MATLAB 2023a environment (Academic Version; MathWorks, Natick, MA, USA), utilizing the COBRA (v2.37.3) and RAVEN (v2.9.2) toolboxes.

### 2.3. Differential Metabolic Analysis via Flux Sampling

#### 2.3.1. Flux Sampling

Based on the constructed host-CRC associated gut microbiota co-metabolic network model, we first defined two key types of transport reactions: (1) Host Transport Reactions (Host_IEX_met[u]tr), responsible for transporting metabolites from host cells to the shared luminal space([u]); and (2) Gut Microbiota Transport Reactions (GM_IEX_met[u]tr), responsible for metabolite uptake from the lumen to the bacterial extracellular space ([e]). Through these two types of transport reactions, we identified the co-metabolites exchanged between the host and the gut microbiota. In this study, a “co-metabolite” is specifically defined as a molecule commonly involved in the metabolic process through the defined transport reactions (i.e., host transport to the lumen and bacterial uptake from the lumen), serving as a crucial molecular mediator of host–microbiota interaction.

To simulate the metabolic environment resulting from host loss-of-function mutations, we constrained the uptake reaction fluxes of specific co-metabolites within bacterial GEMs to zero (lb = ub = 0). This “zero-flux” setting aims to mimic pathological states where the host completely loses the capacity to supply specific substrates to the gut lumen due to gene silencing or transporter inactivation. Notably, as this study focuses on identifying the regulatory effects of co-metabolites, we employed a binary constraint strategy to evaluate trends in bacterial biomass, rather than simulating continuous concentration gradients based on varying host gene expression levels.

Under these constraints, we employed the OptGpSampler algorithm to conduct flux sampling on individual bacterial models. By systematically comparing flux distributions before and after co-metabolite restriction, we aimed to elucidate how host-genetic-driven metabolic changes regulate gut bacteria. OptGpSampler is designed for the rapid, uniform sampling of metabolic solution spaces. It leverages the Artificial Centering Hit-and-Run (ACHR) algorithm to perform centralized sampling within irregular, high-dimensional spaces, enhancing both efficiency and accuracy. All computations were performed using the COBRA Toolbox (v2.37.3) coupled with the Gurobi solver (v11.0.2; Academic License; Gurobi Optimization, LLC, Beaverton, OR, USA).

To ensure the quality and statistical power of the sampled data, we configured the key parameters in accordance with standard practices for the algorithm. First, a thinning factor of 100 was applied to eliminate autocorrelation between adjacent sampling points during the random walk, thereby ensuring that the samples satisfy the independence assumption required for subsequent statistical analyses. Second, 10,000 valid sampling points were extracted from the solution space for each model. This sample size was selected based on previous benchmark studies regarding the convergence of flux sampling in metabolic networks [42,43]. These studies demonstrate that, for GEMs, 10,000 independent sampling points are sufficient to achieve convergence of the flux probability distribution at an acceptable computational cost, allowing for the robust reconstruction of the Probability Density Function of the high-dimensional solution space. Consequently, this sampling scale has been widely adopted in similar systems biology studies to ensure the reliability of metabolic phenotype predictions.

#### 2.3.2. Metabolic Differential Analysis

Based on the flux sampling results, a detailed analysis of the metabolic reaction flux distributions in the CRC-associated gut bacterial GEMs was conducted under the two conditions: co-metabolite-limited and unrestricted (Supplementary Figure S3). Initially, we employed the two-sample Kolmogorov–Smirnov (KS) test (implemented in SciPy v1.14.1) to evaluate the distributional difference between the two sets of flux data. Using a significance level of α = 0.05, metabolic reactions with statistically significant differences in flux distribution (*p*-values < 0.05) were filtered to exclude errors arising from random fluctuation. To further enhance the reliability of the findings, all *p*-values were subjected to False Discovery Rate (FDR) correction using the Benjamini–Hochberg method (implemented in statsmodels v0.14.4).

Subsequently, the magnitude of flux change for each metabolic reaction was quantified using Python (v3.11; Python Software Foundation, Wilmington, DE, USA) (with NumPy v2.1.3 and Pandas v2.2.3 for data processing and calculation. Specifically, the mean flux for each reaction within the CRC-associated bacterial GEMs was calculated for both the limited and unrestricted states, and the normalized flux change (FC) for each metabolic reaction was calculated as follows:

(3) $$\mathrm{FC}=\frac{S_{\mathrm{Restricted}}-S_{\mathrm{Unrestricted}}}{\left|S_{\mathrm{Restricted}}+S_{\mathrm{Unrestricted}}\right|},$$

where $S_{\mathrm{Restricted}}$ and $S_{\mathrm{Unrestricted}}$ represent the average flux of a given metabolic reaction under restricted and unrestricted co-metabolite states, respectively. In this study, we established a stringent FC threshold of 0.82 to screen for significant reactions. This value mathematically corresponds to approximately a 10-fold difference in flux magnitude. This threshold was selected to define “biologically significant” changes, aiming to distinguish dramatic metabolic switch effects from minor steady-state fluctuations commonly observed in GEM [44,45]. By focusing on these large-amplitude changes, we aimed to identify the most robust co-metabolic relationships that drive host–microbiota interactions. It is noteworthy that for reactions present in only one model, their flux in the missing model was assumed to be zero. Furthermore, we employed the Bootstrap (implemented in SciPy v1.14.1) method to estimate 95% confidence intervals (CI) of the flux distributions. If zero did not fall within its 95% CI, the reaction was considered to have significant flux changes and was included in subsequent analyses.

Following the statistical filtering and quantification of metabolic reactions with significant flux changes, we further mapped these differential results to a functional level. Based on the list of reactions identified as having significantly altered fluxes, we employed the Hypergeometric Test (implemented in statsmodels v0.14.4) to perform enrichment analysis on the metabolic subsystems of the 12 CRC-associated bacterial GEMs, aiming to identify metabolic pathways that were significantly enriched and affected by co-metabolite limitation. During the testing process, the probability P(*X* = *k*) that a specific metabolic subsystem contains exactly k significantly changed reactions was calculated using the following formula:

(4) P(X=k)=KkN−Kn−kNn,

where K is the total number of reactions in that metabolic subsystem, N is the total number of all reactions in the entire model, and n is the number of reactions with a significant flux change. When P(*X* = *k*) < 0.05, it indicates that this metabolic subsystem is significantly enriched for the altered reactions, suggesting the effect is more likely driven by co-metabolite limitation rather than random statistical fluctuation. To enhance the reliability of the results, all *p*-values for the metabolic subsystems were also adjusted for FDR using the Benjamini–Hochberg method (statsmodels v0.14.4). Finally, co-metabolites capable of significantly affecting the biomass of the CRC gut bacteria were defined as Key Co-metabolites.

## 3. Results

### 3.1. Quality Assessment of Host and Gut Bacterial GEMs

This study conducted a systematic assessment of the tissue-specificity and quality of the constructed models. Structurally, compared with the generic Human-GEM v1.18, ColonSpecialGEM showed a 16.4% reduction in gene count and a 30.2% decrease in the number of metabolic reactions; the proportion of reactions with gene-protein-reaction (GPR) associations increased to 77.6% (from 62.1% originally). Functionally, FVA demonstrated that the flux span of reactions in ColonSpecialGEM was significantly narrower than that of the generic model (Supplementary Figure S1), and the Wilcoxon signed-rank test confirmed that the solution space contraction was statistically significant (*p* < 6.7 × 10^−6^). Furthermore, regarding reaction essentiality, the tissue-specific constraints revealed 111 essential reactions required for cellular maintenance in ColonSpecialGEM compared to 93 in the generic Human-GEM, highlighting 18 distinct metabolic dependencies specific to the colon tissue environment.

**Figure 1:**
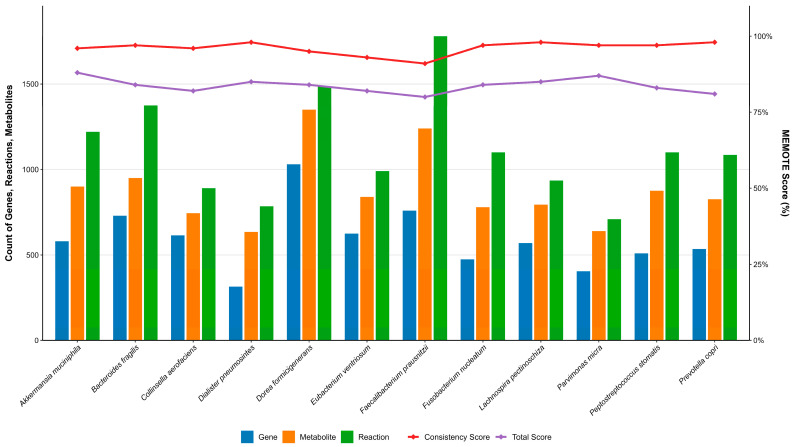
Metabolic information and MEMOTE Test Results of Gut Bacteria GEMs. The bottom bar chart quantitatively represents the architectural complexity of each bacterial GEM, with blue, orange, and green bars depicting the respective counts of genes, reactions, and metabolites. Concurrently, the top line chart provides a qualitative assessment of model quality; the red line illustrates the stoichiometric consistency score, while the purple line denotes the comprehensive MEMOTE score.

Regarding overall quality, ColonSpecialGEM achieved a MEMOTE score of 0.80, while the 12 bacterial GEMs scored between 0.80 and 0.88. All models maintained high stoichiometric consistency scores (0.916–0.995) (Figure 1), collectively confirming that these models met the quality benchmarks required for subsequent simulations in terms of both reaction completeness and metabolic annotation [46].

### 3.2. Structure of Host–Gut Microbiota Co-Metabolic Networks

**Figure 2:**
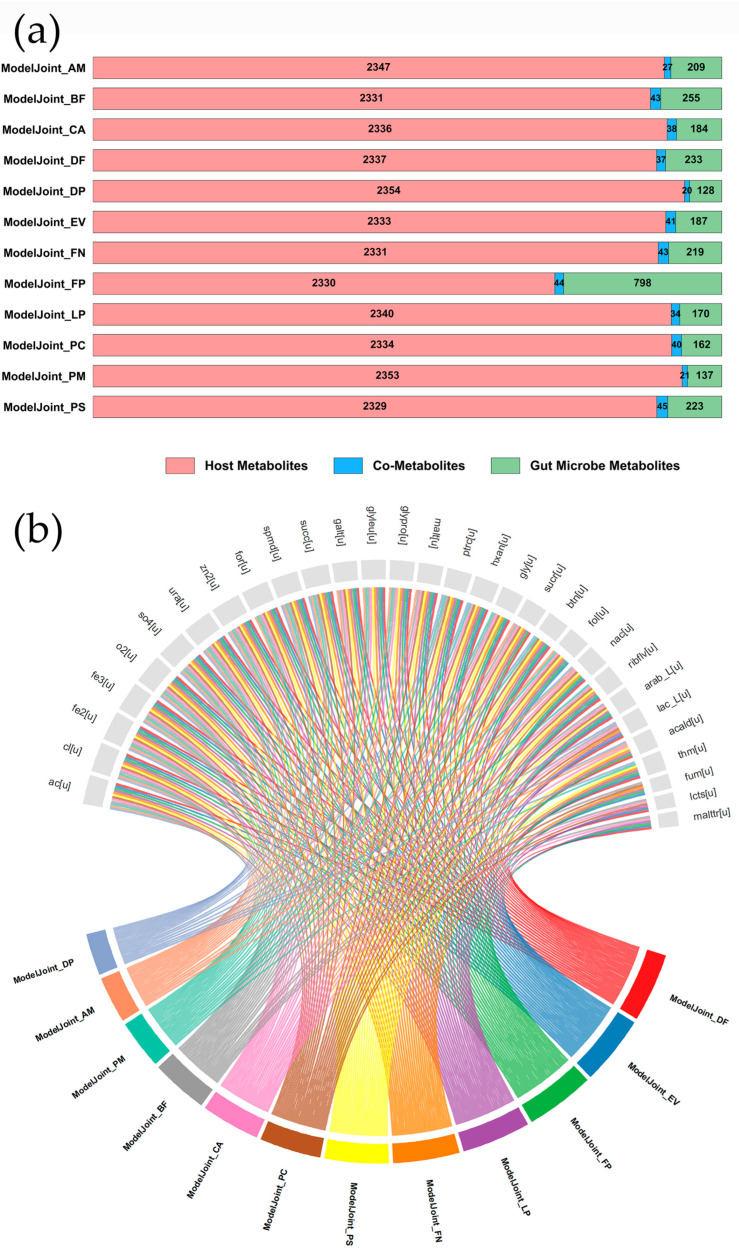
Metabolite Information in the Lumen of Co-Metabolic Network Models. (**a**) Classification of metabolites. The bars display the counts of co-metabolites (blue, exchangeable between host and bacteria), host-specific metabolites (pink), and microbial-specific metabolites (green) within the shared luminal compartment. (**b**) Hierarchical clustering of co-metabolites. Metabolites are ordered by their occurrence frequency across models (decreasing from left to right), with darker colors indicating presence.

ColonSpecialGEM was integrated with 12 CRC-associated gut bacterial GEMs to successfully construct host–microbiota co-metabolic network models. Given the pivotal role of co-metabolites as crucial mediators linking host and microbial metabolic interactions, a systematic analysis was conducted. The analysis of the co-metabolic network structure initially revealed the dominant role of the host in intestinal metabolism (Figure 2a): results indicated that each co-metabolic network comprised 20–45 co-metabolites, with host-derived metabolites accounting for a remarkably high proportion, ranging from 82.1% to 91.3%, significantly exceeding the contribution from microbial sources. Furthermore, the distribution of co-metabolites exhibited a distinct hierarchical characteristic (Figure 2b), with seven co-metabolites (including acetate, chloride ion, ferric iron, ferrous iron, zinc ion, sulfate, and uracil) universally present across all 12 models. Subsequently, metabolites such as L-arabinose, L-lactate, and thiamin were identified as next most frequently occurring co-metabolites, detected in 8–9 co-metabolic models.

### 3.3. Screening of Key Co-Metabolites

**Figure 3:**
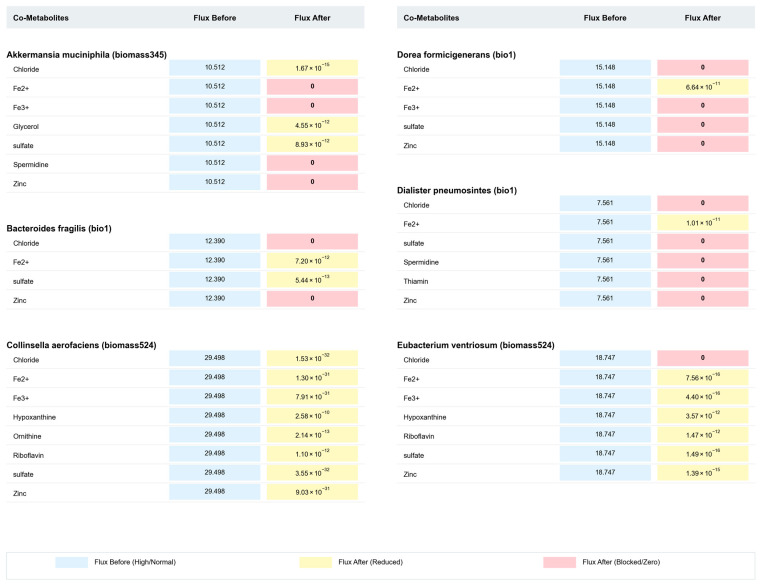
Comparative analysis of biomass reaction flux for six bacterial strains (Part I). This figure displays the flux changes before and after co-metabolite restriction for *Akkermansia muciniphila*, *Bacteroides fragilis*, *Collinsella aerofaciens*, *Dorea formicigenerans*, *Dialister pneumosintes*, *and Eubacterium ventriosum*. The heatmap uses color gradients to represent flux changes: blue indicates high/normal flux (Flux Before), yellow indicates reduced flux (Flux After—Reduced), and pink indicates blocked/zero flux (Flux After—Blocked/Zero).

**Figure 4:**
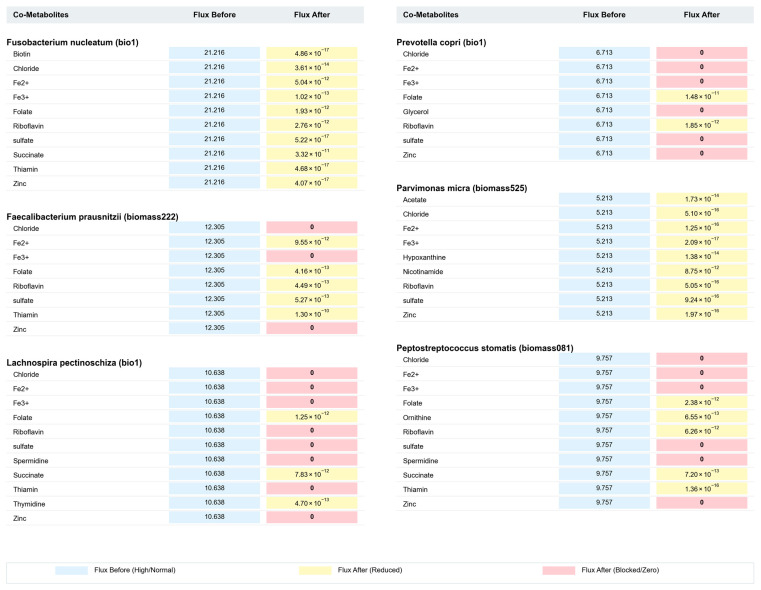
Comparative analysis of biomass reaction flux for six bacterial strains (Part II). This figure displays the flux changes before and after co-metabolite restriction for *Fusobacterium nucleatum*, *Faecalibacterium prausnitzii*, *Lachnospira pectinoschiza*, *Prevotella copri*, *Parvimonas micra*, *and Peptostreptococcus stomatis*. The color coding follows the same scheme as Figure 3: blue indicates high/normal flux, yellow indicates reduced flux, and pink indicates blocked/zero flux.

To precisely identify molecules from the co-metabolite pool that exert critical regulatory effects on gut bacterial growth, this study employed a comprehensive strategy combining metabolic flux sampling with differential metabolic analysis. We identified 17 “key co-metabolites” based on the criterion that their restricted uptake leads to a significant reduction in bacterial biomass flux (Table 2). Figure 3 and Figure 4 illustrate the changes in biomass reaction flux for 12 strains under these key co-metabolite limitation conditions. The results demonstrate that, under the influence of key co-metabolite restriction, the biomass reaction flux of all 12 CRC-associated gut bacteria significantly decreased, with some strains exhibiting a complete cessation of growth.

To further validate the clinical relevance of these in silico predictions, we systematically cross-referenced these metabolites with the Human Metabolome Database (HMDB) and existing clinical metabolomics literature (Table 3). This comparison revealed a strong qualitative correlation between our predicted metabolic dependencies and reported dysregulation patterns in CRC patients.

Among the organic metabolites, Acetate and Succinate were highlighted. Acetate, a representative SCFA, serves not only as an energy substrate but also acts as a signaling molecule that modulates the gut microbiome and glycolysis pathways, thereby influencing CRC therapeutic efficacy [56]. Similarly, Succinate functions as a critical metabolic intermediate; notably, *Fusobacterium nucleatum*-derived succinic acid has been shown to induce tumor resistance to immunotherapy, underscoring its pivotal role in CRC progression [57].

Regarding inorganic nutrients, Ferric iron, Chloride, Zinc, and Sulfate were identified as core regulatory factors. Iron is essential for bacterial proliferation but also participates in the Fenton reaction, generating reactive oxygen species (ROS); recent evidence suggests that dietary iron significantly modulates gut microbiota composition to promote colorectal tumorigenesis [58]. Furthermore, inorganic ions were consistently identified across all 12 network models, likely regulating bacterial survival through fundamental physiological mechanisms—such as Chloride-mediated osmotic pressure regulation and membrane potential maintenance.

### 3.4. Impact of Key Co-Metabolites on Gut Bacterial Metabolic Subsystems

In order to further investigate the specific molecular mechanisms by which key co-metabolites influence strain biomass, this study, utilizing 12 gut microbial GEMs, identified affected metabolic subsystems by statistically analyzing significantly altered metabolic reactions following key metabolite limitations, combined with hypergeometric enrichment analysis.

**Figure 5:**
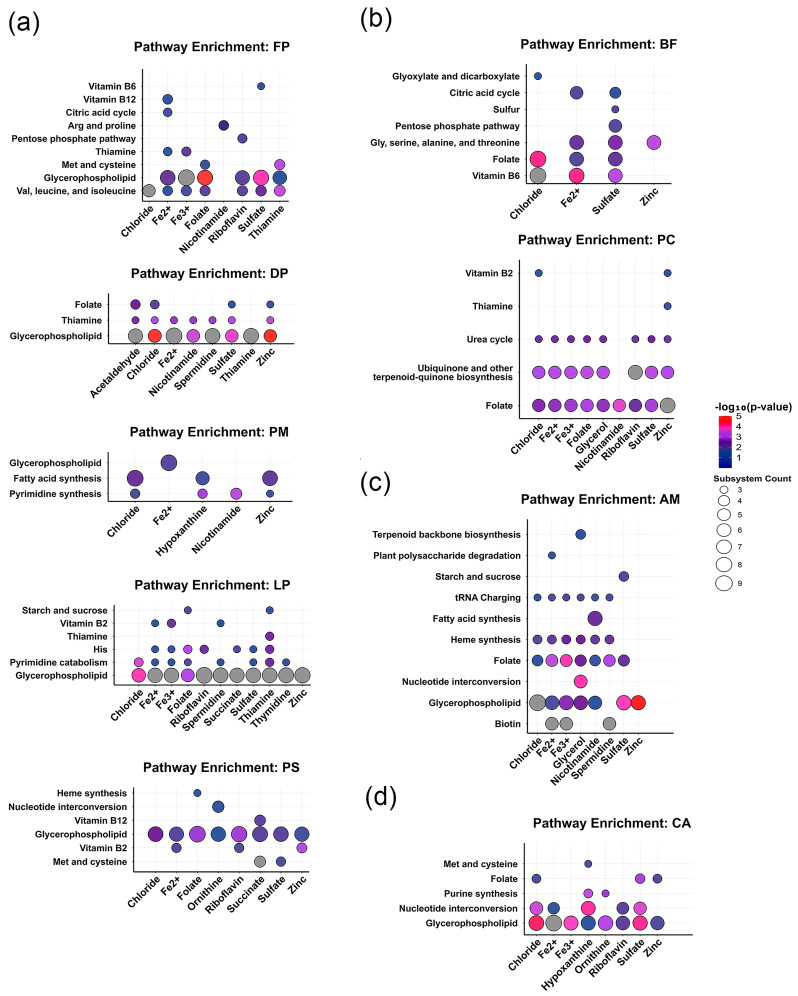
Significantly Altered Metabolic Subsystems in the GEM Metabolic Difference Analysis of CRC-Associated Gut Bacteria. This figure comprehensively illustrates the impact of restricting 17 key co-metabolites on the metabolic networks of a total of 9 CRC-associated gut bacterial strains from four distinct phyla. The x-axis represents the key co-metabolites, and the y-axis indicates the related metabolic reaction pathways (subsystems). Each panel displays: (**a**) Five gut bacterial strains from the Firmicutes phylum; (**b**) Two gut bacterial strains from the Bacteroidetes phylum; (**c**) One gut bacterial strain from the Verrucomicrobia phylum; (**d**) One gut bacterial strain from the Actinobacteria and Fusobacteria phyla.

The hypergeometric enrichment analysis of metabolic subsystems in five CRC-associated gut bacteria from the phylum Firmicutes (Figure 5a) revealed that glycerophospholipid metabolism was the most significantly affected metabolic pathway by key co-metabolites. In five of these strains, namely *Dialister pneumosintes*, *Faecalibacterium prausnitzii*, *Lachnospira pectinoschiza*, *Parvimonas micra*, and *Peptostreptococcus stomatis*, this pathway was significantly modulated by a variety of key co-metabolites, including chloride ions, ferric ions, ferrous ions, thiamine, folate, and nicotinamide. Compared to other metabolic subsystems, the glycerophospholipid metabolism pathway exhibited the highest number of enriched metabolic reaction changes. Furthermore, thiamine metabolism was also influenced by multiple key co-metabolites in *D. pneumosintes*, *F. prausnitzii*, and *L. pectinosc-hiza*.

Metabolic responses of Bacteroides fragilis and *Prevotella copri* from the phylum Bacteroidetes exhibited distinct functional differentiation under key co-metabolite limitation (Figure 5b). Metabolic subsystem changes in B. fragilis primarily concentrated on vitamin metabolism and amino acid metabolism. In terms of vitamin metabolism, its vitamin B6 and folate metabolism pathways were significantly affected by three inorganic ion key co-metabolites: chloride ions, ferrous ions, and sulfate. Regarding amino acid metabolism, the glycine-serine-alanine-threonine metabolism pathway was influenced by ferrous ions, sulfate, and zinc ions as key co-metabolites. In contrast, metabolic changes in P. copri mainly focused on energy metabolism and nitrogen cycling, where ubiquinone and other terpenoid-quinone biosynthesis pathways, as well as the urea cycle pathway, were broadly regulated by various key co-metabolites including chloride ions, ferric ions, folate, glycerol, nicotinamide, and riboflavin.

Under the influence of key co-metabolites, *Akkermansia muciniphila* from the phylum *Verrucomicrobia* exhibited complex metabolic changes (Figure 5c). This bacterium’s glycerophospholipid metabolism and folate metabolism pathways demonstrated high sensitivity to various key co-metabolites, including five inorganic ions (chloride, ferrous, ferric, sulfate, and zinc ions), as well as glycerol, nicotinamide, and spermidine. In addition to these pathways, eight other secondary metabolic pathways, such as biotin metabolism, were synergistically regulated to varying degrees by these metabolites.

Similar to other phyla of CRC-associated gut bacteria, the glycerophospholipid metabolism pathway in *Collinsella aerofaciens* from the phylum *Actinobacteria* also demonstrated a significant dependence on various inorganic ions and key co-metabolites such as hypoxanthine, ornithine, and riboflavin (Figure 5d). Notably, hypoxanthine significantly impacted multiple metabolic subsystems in this strain, including purine synthesis, nucleotide interconversion, methionine and cysteine metabolism, and glycerophospholipid metabolism.

## 4. Discussion

By integrating a colon tissue-specific host genome-scale metabolic model with multiple CRC-associated bacterial models, this study establishes a causal “host-metabolite-gut microbiota-CRC” regulatory axis. Notably, the methodology for constructing host–microbiota co-metabolic models has been successfully applied in various studies, encompassing inter-microbial metabolic interactions within the gut microbiota and host–gut microbiota metabolic interactions in murine models [59,60]. We reveal that a remarkably defined set of host-supplied metabolites functions as the central control hub linking genetic susceptibility to microbial dysbiosis and colorectal carcinogenesis.

The dominance of host-derived metabolic fluxes in our co-metabolic networks confirms the host’s role as the primary architect of the intestinal nutrient landscape [61]. This implies that even minor, genetically driven impairments in host synthesis or transport capacity can profoundly reshape microbial community structure. Consequently, this provides a mechanistic explanation for how early dysbiosis can emerge in genetically predisposed individuals, even before the onset of overt pathology.

While hundreds of compounds are exchanged at the host-microbe interface, our findings demonstrate that the growth of CRC-associated strains critically depends on a limited repertoire of “bottleneck metabolites.” Unlike simple nutrients, these identified co-metabolites (including SCFAs, iron, and specific vitamins) act as dual-functional regulators: they not only strictly govern bacterial biomass synthesis but also directly modulate tumor biology. As validated by recent clinical literature, the host’s inability to maintain homeostatic levels of these compounds—such as the depletion of protective acetate or the accumulation of pro-tumorigenic succinate and iron—simultaneously favors the expansion of pathogenic taxa and creates a permissive microenvironment for tumorigenesis [47,48,49,51]. This dual-control mechanism highlights these metabolites as high-value targets for precision intervention.

Furthermore, we observed distinct phylum-specific metabolic reprogramming strategies in response to these co-metabolite limitations. The divergent adaptation patterns—such as the broad sensitivity of Firmicutes in lipid and redox pathways versus the specialized amino acid salvage pathways in Bacteroidetes—reflect evolutionarily shaped nutritional niches [62,63,64]. These specific metabolic signatures explain why identical host metabolic defects (e.g., iron or folate transport deficiencies) do not affect the microbiome uniformly but instead drive a selective ecological shift that favors opportunistic pathogens while eliminating beneficial commensals, ultimately precipitating the dysbiosis characteristic of CRC.

Genetically anchoring this regulatory axis, we identified 78 host genes responsible for the transport and synthesis of these critical co-metabolites, with a significant proportion (~30%) overlapping with established CRC risk loci (Supplementary Figure S5 and Table S1). Gene Ontology (GO) enrichment analysis revealed significant enrichment of these genes in fundamental metabolic processes such as “organic acid transport,” “metal ion homeostasis,” and “vitamin metabolism,” indicating that the host maintains intestinal microbial homeostasis through precise regulation of these substance transports (Supplementary Figure S6). The functional enrichment of these genes in pathways related to “anticancer drug resistance” is particularly noteworthy (Supplementary Figure S7). It suggests that common or rare genetic variants may impair the supply of key co-metabolites, thereby provoking selective dysbiosis that contributes not only to CRC initiation but also to therapy resistance. This systematically demonstrates that the synthesis and transport of key co-metabolites are highly likely to directly influence CRC pathological processes through host–microbiota interactions, confirming the robust clinical potential of these metabolic transporter genes in modulating CRC progression and overcoming anticancer drug resistance.

The present framework unifies multiple previously disconnected CRC risk factors into a single actionable model. Well-known high-risk polymorphisms, such as those in *Nucleotide-binding Oligomerization Domain-containing protein 2* (NOD2) and *Fucosyl-transferase 2* (FUT2), likely converge on the bottleneck metabolites identified here—specifically by modulating the availability of nutrients like iron and folate [65,66]. This creates a permissive environment for “driver” bacteria: *Fusobacterium nucleatum* thrives via succinate-dependent immune evasion [60], while enterotoxigenic Bacteroides fragilis exploits sulfate metabolism to fuel proliferation [67,68]. Moreover, the link between host transporter genes (e.g., chloride and zinc transporters) and chemotherapy resistance offers a novel mechanistic angle on microbiota-mediated treatment failure [69]. Consequently, genotype-guided interventions—such as targeted folate/acetate supplementation or dietary iron restriction—emerge as promising metabolism-centered strategies for precision CRC prevention and adjuvant therapy.

Despite these mechanistic insights, this study has several limitations that should be acknowledged. First, regarding model specificity, ethical and data constraints compelled us to use healthy human transcriptomic data for the host GEM rather than CRC-altered tissue. While this means the model cannot fully capture the Warburg effect or metabolic reprogramming characteristic of advanced tumors, this design was intentional to capture the “initiation phase” of dysbiosis driven by host genetic susceptibility prior to overt tumorigenesis. Second, regarding microbial complexity, the co-metabolic network adopts a “host-single strain” strategy. We acknowledge that this reductionist approach ignores inter-microbial competition and cross-feeding. However, this simplification allows for the unambiguous identification of host-driven metabolic bottlenecks without the confounding noise of complex ecological dynamics, serving as a necessary first step in decoding host control. Third, regarding regulatory directionality, the current model focuses on “top-down” regulation from host to microbiota. It does not explicitly model metabolic feedback loops from the microbiota to the host, which are known to influence host physiology. Future work must integrate bidirectional signaling to establish a more dynamic system model. Finally, regarding methodological constraints, while we employed standard flux sampling parameters (e.g., thinning factor = 100) validated by benchmark studies to ensure convergence, we did not perform a comprehensive sensitivity analysis for all sampling parameters. Consequently, the results should be interpreted as robust predictions of metabolic potential rather than precise quantitative measurements of in vivo fluxes.

In conclusion, by moving beyond statistical associations to systems-level metabolic mechanisms, this work positions a genetically controlled, host-derived metabolite bottleneck as the central node integrating hereditary susceptibility, microbial dysbiosis, and colorectal tumorigenesis. These findings provide a solid mechanistic foundation for the development of next-generation metabolite-based diagnostic biomarkers and therapeutic interventions.

## 5. Conclusions

In this study, we successfully elucidated the critical metabolic interaction mechanisms between the host and gut microbiota in CRC through the construction of a co-metabolic network model, integrating a host colon tissue-specific GEM with 12 CRC pro-/anti-carcinogenic gut bacterial GEMs. We identified 17 key co-metabolites, including chloride, zinc ions, and acetate, that significantly regulate bacterial biomass, with 13 of these being validated by literature as associated with colorectal cancer. Furthermore, we deeply clarified the molecular mechanisms by which these co-metabolites primarily influence microbial function and biomass by acting upon core metabolic pathways such as glycerophospholipid metabolism and folate metabolism. The findings of this research provide a systematic perspective and mechanistic insight into how the host’s genetic background, through the modulation of co-metabolites, impacts gut microbiota and ultimately influences CRC progression.

## Figures and Tables

**Table 1:** Twelve Literature-Validated CRC-Associated Gut Bacteria with Pro- and Anti-tumorigenic Functions.

Bacterial Species	Phenotype in CRC	References/Project ID
AM	Inhibiting	[26], PRJNA397219
BF	Promoting	[27,28,29], PRJEB10878
CA	Inhibiting	[30], PRJNA397219
DP	Promoting	[31], PRJDB4176, PRJEB10878
DF	Inhibiting	[27,32]
EV	Inhibiting	[28], PRJEB10878
FP	Inhibiting	[33,34], PRJEB10878
FN	Promoting	[35,36,37], PRJEB10878, PRJNA397219
LP	Inhibiting	[32], PRJEB10878
PM	Promoting	[27,31] PRJEB10878, [27], PRJNA397219
PS	Promoting	[31], PRJDB4176, PRJEB10878
PC	Inhibiting	[32], PRJDB4176, PRJNA397219

Gut microbiota abbreviations correspond to: AM (*Akkermansia muciniphila*); BF (*Bacteroides fragilis*); CA (*Collinsella aerofaciens*); DP (*Dialister pneumosintes*); DF (*Dorea formicigenerans*); EV (*Eubacterium ventriosum*); FP (*Faecalibacterium prausnitzii*); FN (*Fusobacterium nucleatum*); LP (*Lachnospira pec-tinoschiza*); PM (*Parvimonas micra*); PS (*Peptostreptococcus stomatis*); PC (*Prevotella copri*).

**Table 2:** Effects of Key Co-Metabolites on the Biomass of CRC-Associated Gut Bacteria.

Key Metabolite	Related Gut Microbiota	Influence of Reaction
Acetate	PM	Reduction
Succinate	FN, LP, PS	Reduction
Chloride	AM, BF, CA, DF, DP, EV, FN, FP, LP, PC, PM, PS	Reduction
Fe^2+^	DF, DP, EV, FN, FP, LP, PC, PM	Reduction
Fe^3+^	AM, CA, DF, EV, FN, FP, LP, PC, PM, PS	Reduction
Zinc	AM, BF, CA, DF, DP, EV, FN, FP, LP, PC, PM, PS	Reduction
Sulfate	AM, BF, CA, DF, DP, EV, FN, FP, LP, PC, PM, PS	Reduction
Glycerol	AM, PC	Reduction
Riboflavin	CA, EV, FN, FP, LP, PC, PM, PS	Reduction
Thiamin	DP, FN, FP, LP, PS	Reduction
Biotin	FN	Reduction
Folate	FN, FP, LP, PC, PS	Reduction
Nicotinamide	PM	Reduction
Spermidine	AM, DP, LP, PS	Reduction
Ornithine	CA, PS	Reduction
Hypoxanthine	CA, EV, PM	Reduction
Thymidine	LP	Reduction

Gut microbiota abbreviations correspond to: AM (*Akkermansia muciniphila*); BF (*Bacteroides fragilis*); CA (*Collinsella aerofaciens*); DP (*Dialister pneumosintes*); DF (*Dorea formicigenerans*); EV (*Eubacterium ventriosum*); FP (*Faecalibacterium prausnitzii*); FN (*Fusobacterium nucleatum*); LP (*Lachnospira pectinoschiza*); PM (*Parvimonas micra*); PS (*Peptostreptococcus stomatis*); PC (*Prevotella copri*).

**Table 3:** Identified Co-metabolic Regulators of CRC Microbiota.

Key Metabolite	HMDB ID	References	Related Evidence
Acetate/Acetic acid	HMDB0000042	[47]	As a key SCFA, Acetate directly inhibits CRC growth and suppresses tumor development by modulating the Wnt/β-catenin pathway.
Succinate/Succinic acid	HMDB0000254	[48]	Acts as a signaling molecule that promotes CRC immuno-evasion and progression by suppressing host cGAS-STING immunity.
Fe^2+^/Fe^3^(Iron)	HMDB0001314	[49]	Dietary Iron promotes CRC tumorigenesis by modulating the gut microbiota and inducing the secretion of specific host factors.
Sulfate	HMDB0001448	[50]	H_2_S, a key sulfur metabolite, promotes the proliferation of CRCs
Hypoxanthine	HMDB0000157	[51]	The metabolite Inosine, a purine derivative, is listed as a gut microbiota metabolite that inhibits CRC.
Chloride	HMDB0000060	[52]	Chloride channels and transporters are involved in the occurrence and development of CRC.
Spermidine	HMDB0001257	[53]	Spermidine overaccumulation is directly associated with tumor cell survival.
Zn/Zinc	HMDB0001303	[54]	Serum zinc levels are significantly decreased in CRC patients.
Riboflavin	HMDB0000244	[55]	High serum riboflavin is associated with the risk of sporadic colorectal cancer
Thiamin	HMDB0000235	[56]	Low-dose thiamine stimulates tumor growth.
Glycerol	HMDB0000131	[57]	Glycerol may serve as a biomarker for CRC and be applied to early diagnostic tools.
Folate/Folic acid	HMDB0000079	[58]	Folic acid-related genes were identified as therapeutic targets for CRC.

## Data Availability

Data available in a publicly accessible repository. The original data, including the customized host and bacterial GEMs and all analysis scripts (developed and executed using MATLAB 2023a with COBRA v2.37.3 and Gurobi v11.0.2), have been deposited in the project’s GitHub repository (https://github.com/wjsjshgsy/Co-metabolic-network-analysis, accessed on 12 December 2025). Full details of the software versions and computing environments are provided in the Materials and Methods section. Further inquiries can be directed to the corresponding author.

## Supplementary Materials

The following supporting information can be downloaded at: https://www.mdpi.com/article/10.3390/metabo16010064/s1, Figure S1: Gut Bacteria-CRC Association Information in MicroPhenoDB, gutMDisorder, and Peryton Databases; Table S1. Twenty-three Validated Genes Associated with CRC; Figure S2: Schematic Diagram of Host-CRC Associated Gut Bacteria Co-Metabolic Model; Figure S3. Metabolic Difference Analysis Workflow of CRC-Associated Gut Bacterias GEM; Figure S4: Comparison of flux variability ranges between the generic Human-GEM and ColonSpecialGEM; Figure S5: Host Genes-Key Co-Metabolites Regulatory Network; Figure S6: GO Enrichment Analysis of Host Genes Related to Key Co-Metabolites; Figure S7: KEGG Pathway Enrichment Analysis of Host Genes Related to Key Co-Metabolites.

## Abbreviations

The following abbreviations are used in this manuscript:

CRC	Colorectal cancer
GEM	Genome-Scale Metabolic Model
SCFAs	Short-chain fatty acids
mGWAS	metabolome-wide genome-wide association study
GWAS	Genome-Wide Association Study
MR	Mendelian randomization
IBD	inflammatory bowel disease
FVA	Flux Variability Analysis
TPM	Transcripts Per Million
KS	Kolmogorov–Smirnov
FDR	False Discovery Rate
PDF	Probability Density Function
FC	flux change
CI	confidence interval
HMDB	Human Metabolome Database
ROS	generating reactive oxygen species
GPR	Gene-Protein-Reaction
GO	Gene Ontology
KEGG	Kyoto Encyclopedia of Genes and Genomes
