# Supplementary material for: Co-Metabolic Network Reveals the Metabolic Mechanism of Host–Microbiota Interplay in Colorectal Cancer
Source: Metabolites. 2026 Jan 11;16(1):64. doi: 10.3390/metabo16010064 (PMC12844167; doi:10.3390/metabo16010064)
Supplement: Supplementary file 1 [file metabolites-16-00064-s001.zip › Table S1.docx]

**Supplementary** **Table S1.** Twenty-three Validated Genes Associated with CRC.

| **Gene** | **CRC Association Type** |
| --- | --- |
| ABCBA | Biomarker |
| ABCC1 | Biomarker, Genetic Variation |
| ABCC2 | Biomarker, Genetic Variation |
| ABCC4 | Biomarker |
| ABCC5 | Biomarker |
| ABCG2 | Biomarker, Genetic Variation |
| ACHE | AlteredExpression |
| BCHE | AlteredExpression |
| CYRBD1 | Biomarker |
| FOLR1 | Biomarker |
| FTH1 | Biomarker |
| GLB1 | Biomarker |
| ODC1 | AlteredExpression, Biomarker, Genetic Variation |
| SLC16A1 | AlteredExpression |
| SLC16A4 | AlteredExpression |
| SLC16A7 | AlteredExpression |
| SLC19A1 | Biomarker, Genetic Variation |
| SLC26A3 | Biomarker |
| SLC39A14 | Biomarker |

Twenty-three genes significantly associated with CRC in the DisGeNET and CTD databases. These genes are primarily involved in CRC pathogenesis through mechanisms such as aberrant expression, functioning as biomarkers, and genetic variation.
