# Supplementary figures and images for: Co-Metabolic Network Reveals the Metabolic Mechanism of Host–Microbiota Interplay in Colorectal Cancer
Source: Metabolites. 2026 Jan 11;16(1):64. doi: 10.3390/metabo16010064 (PMC12844167; doi:10.3390/metabo16010064)

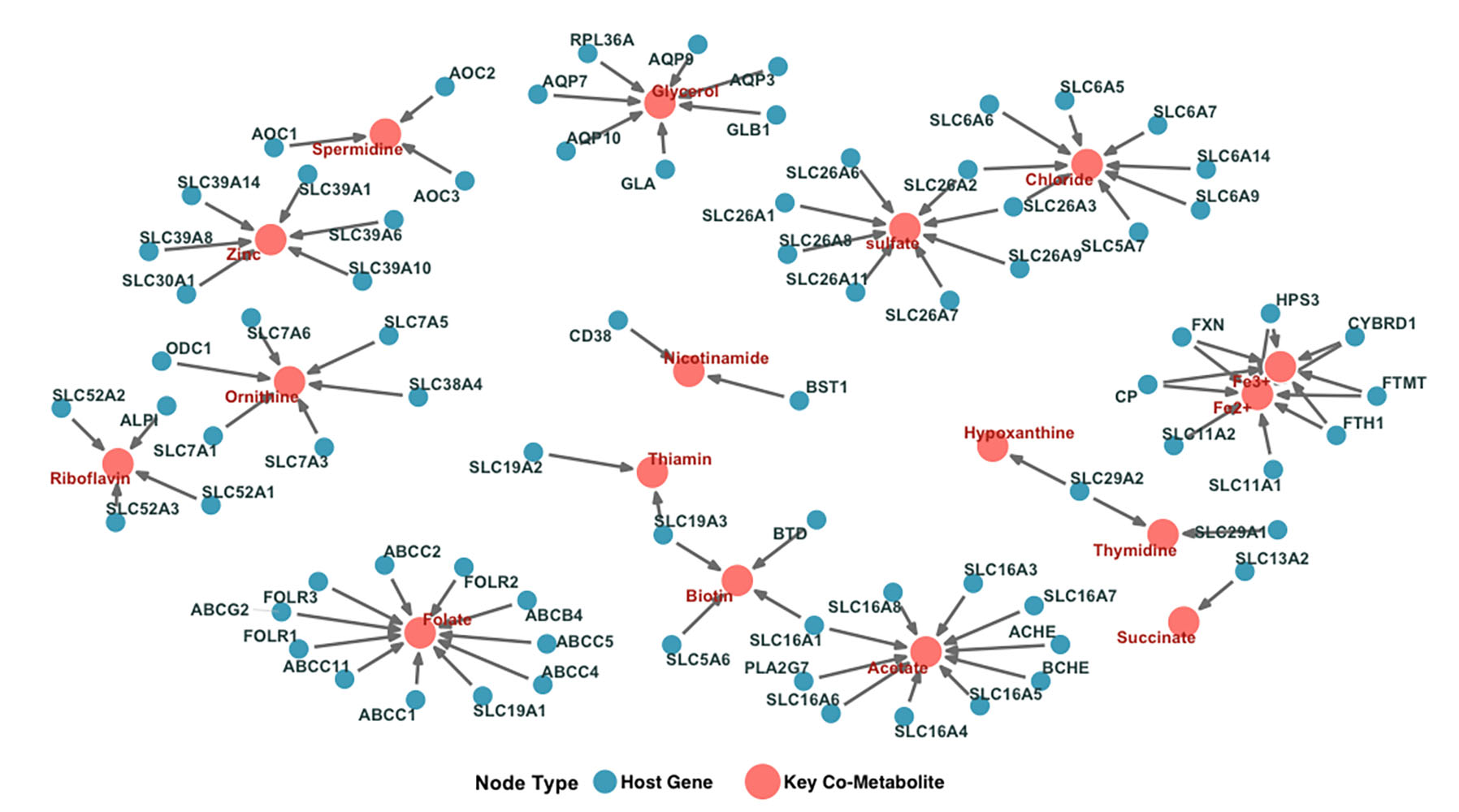

Supplement: Supplementary file 1 [file metabolites-16-00064-s001.zip › Figure S5.jpg]

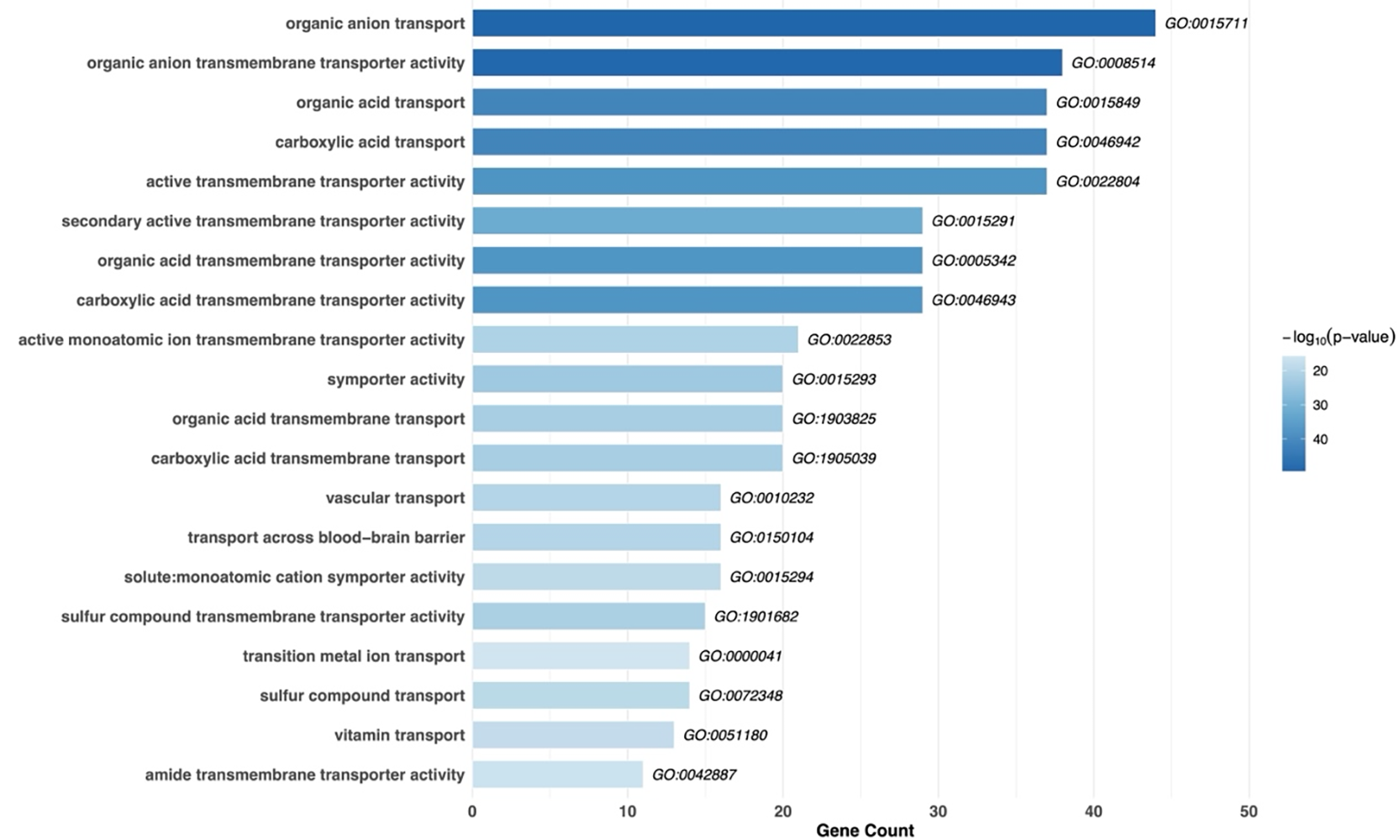

Supplement: Supplementary file 1 [file metabolites-16-00064-s001.zip › Figure S6.png]

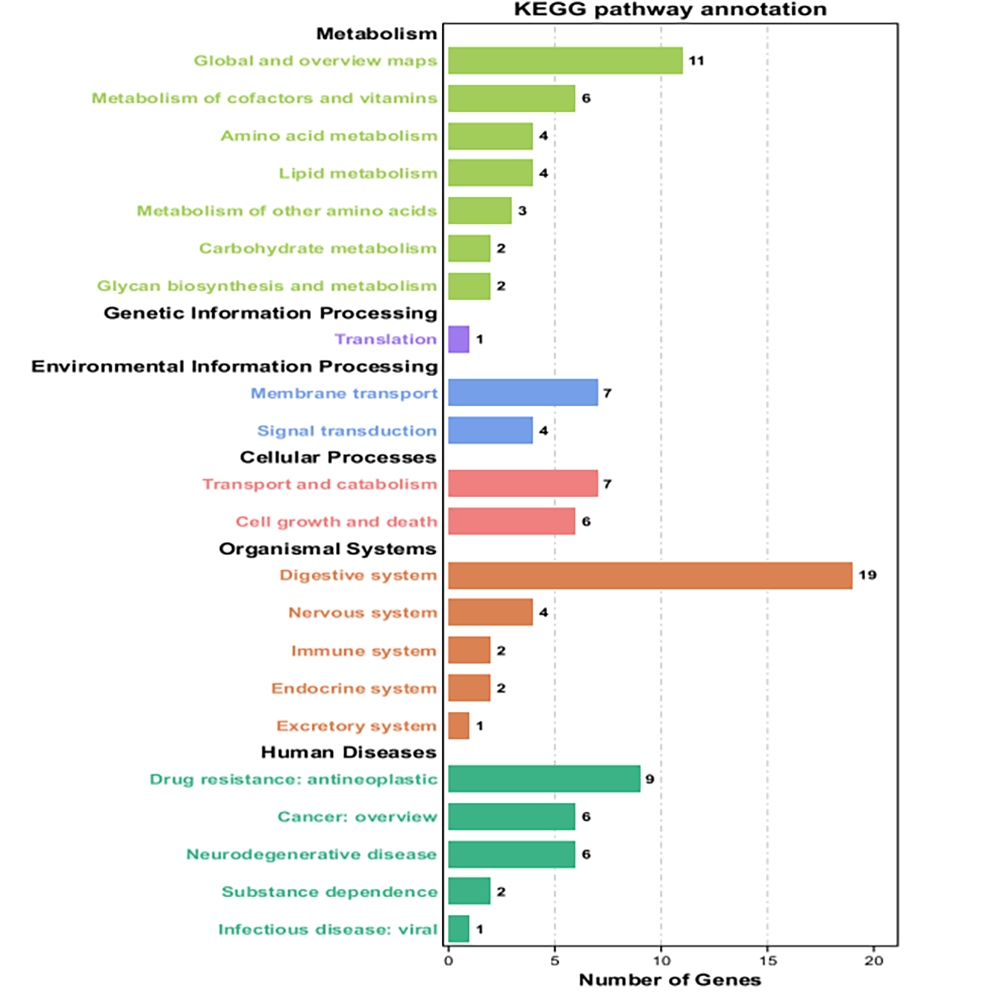

Supplement: Supplementary file 1 [file metabolites-16-00064-s001.zip › Figure S7.png]

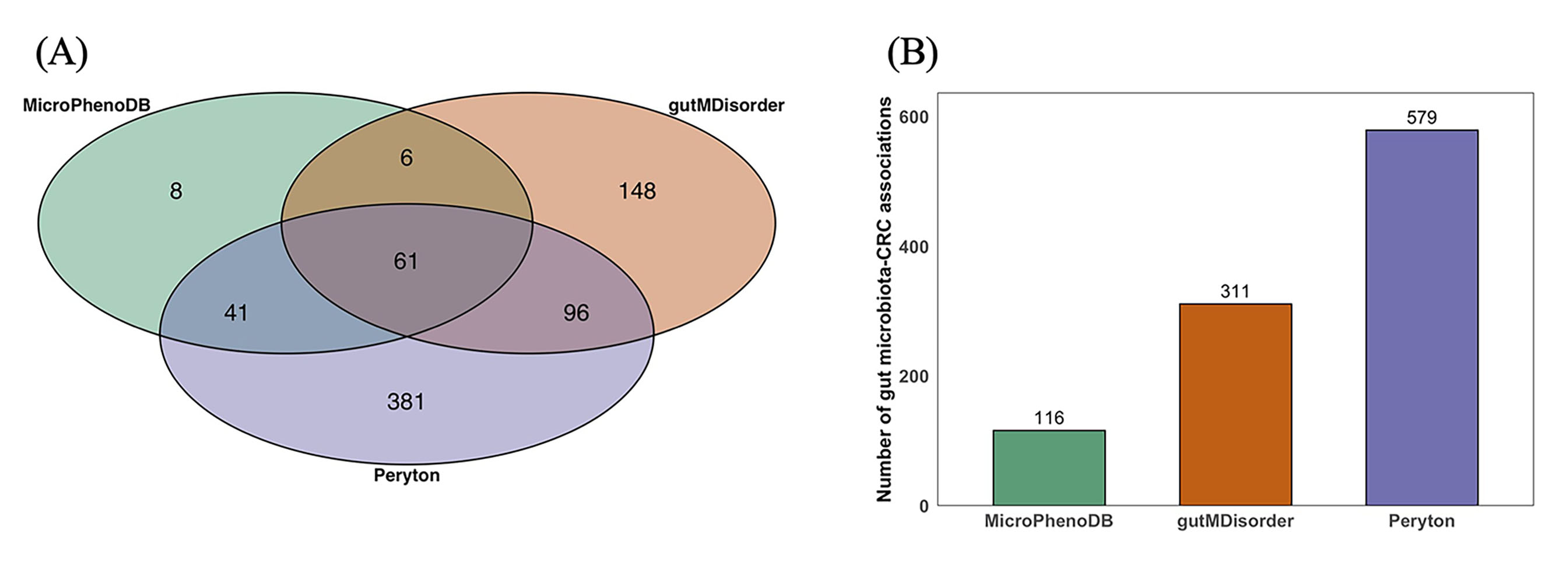

Supplement: Supplementary file 1 [file metabolites-16-00064-s001.zip › Supplementary Figure S1 .jpg]

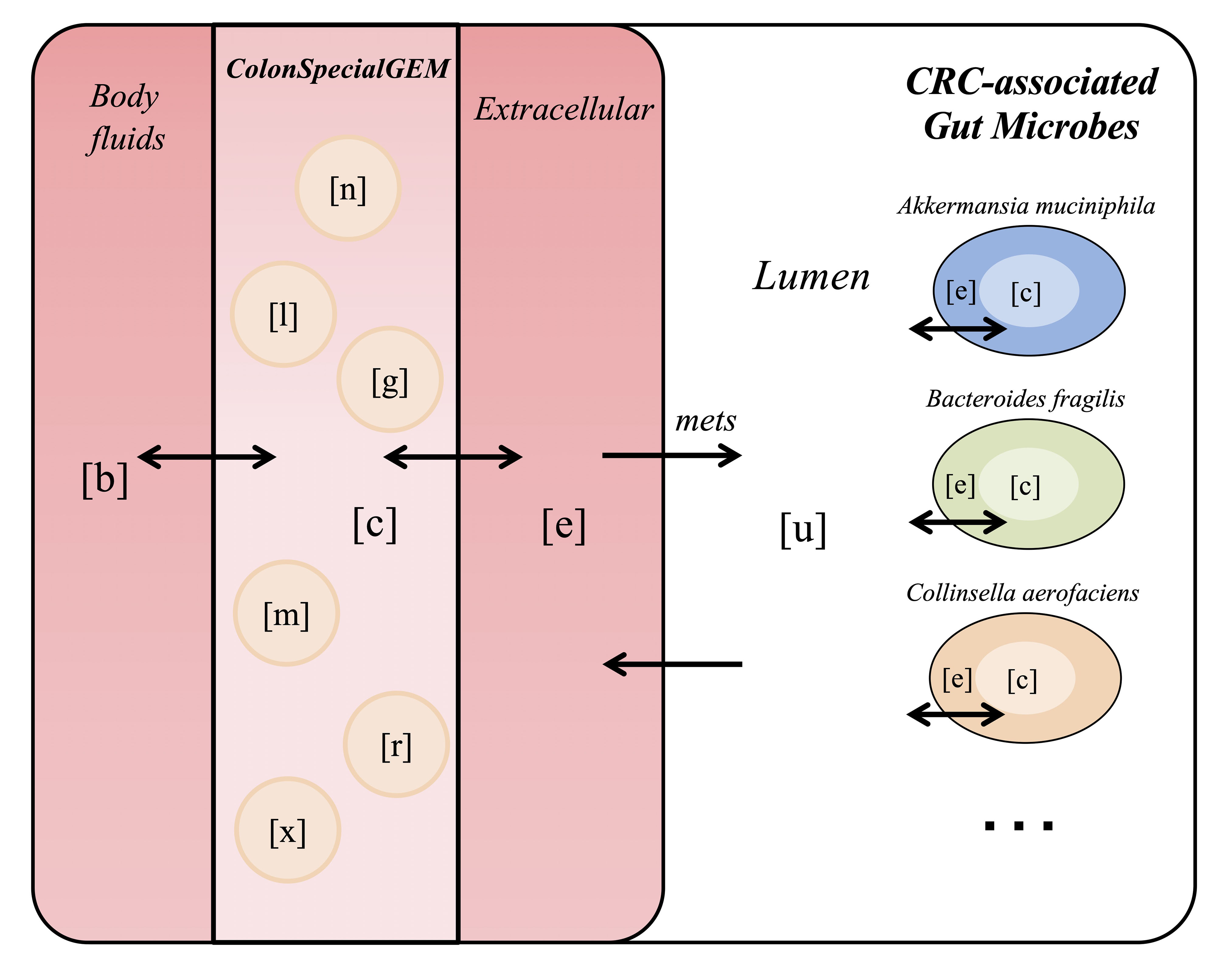

Supplement: Supplementary file 1 [file metabolites-16-00064-s001.zip › Supplementary Figure S2.jpg]

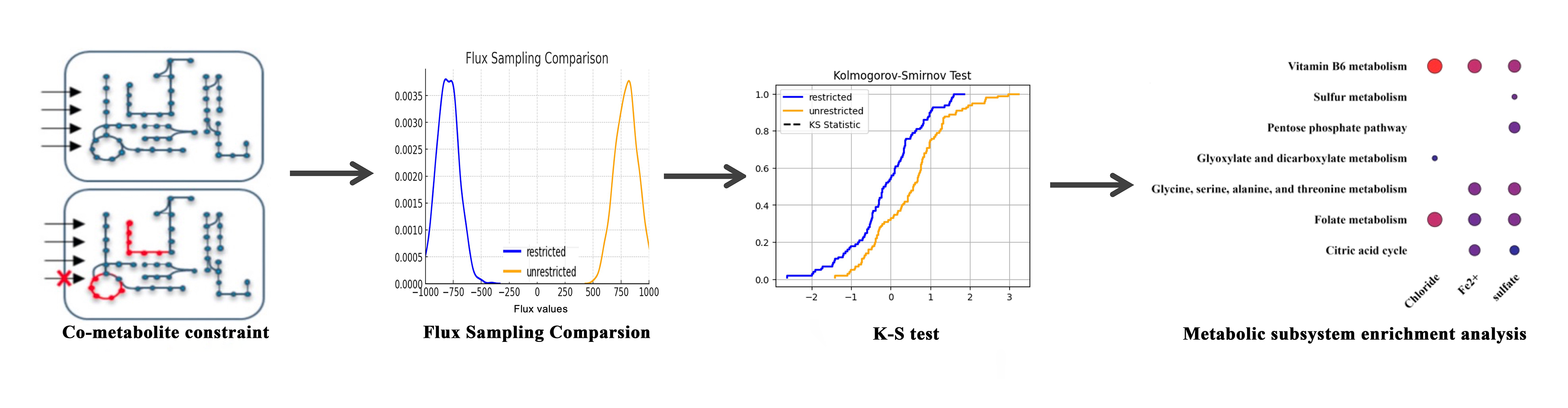

Supplement: Supplementary file 1 [file metabolites-16-00064-s001.zip › Supplementary Figure S3.png]

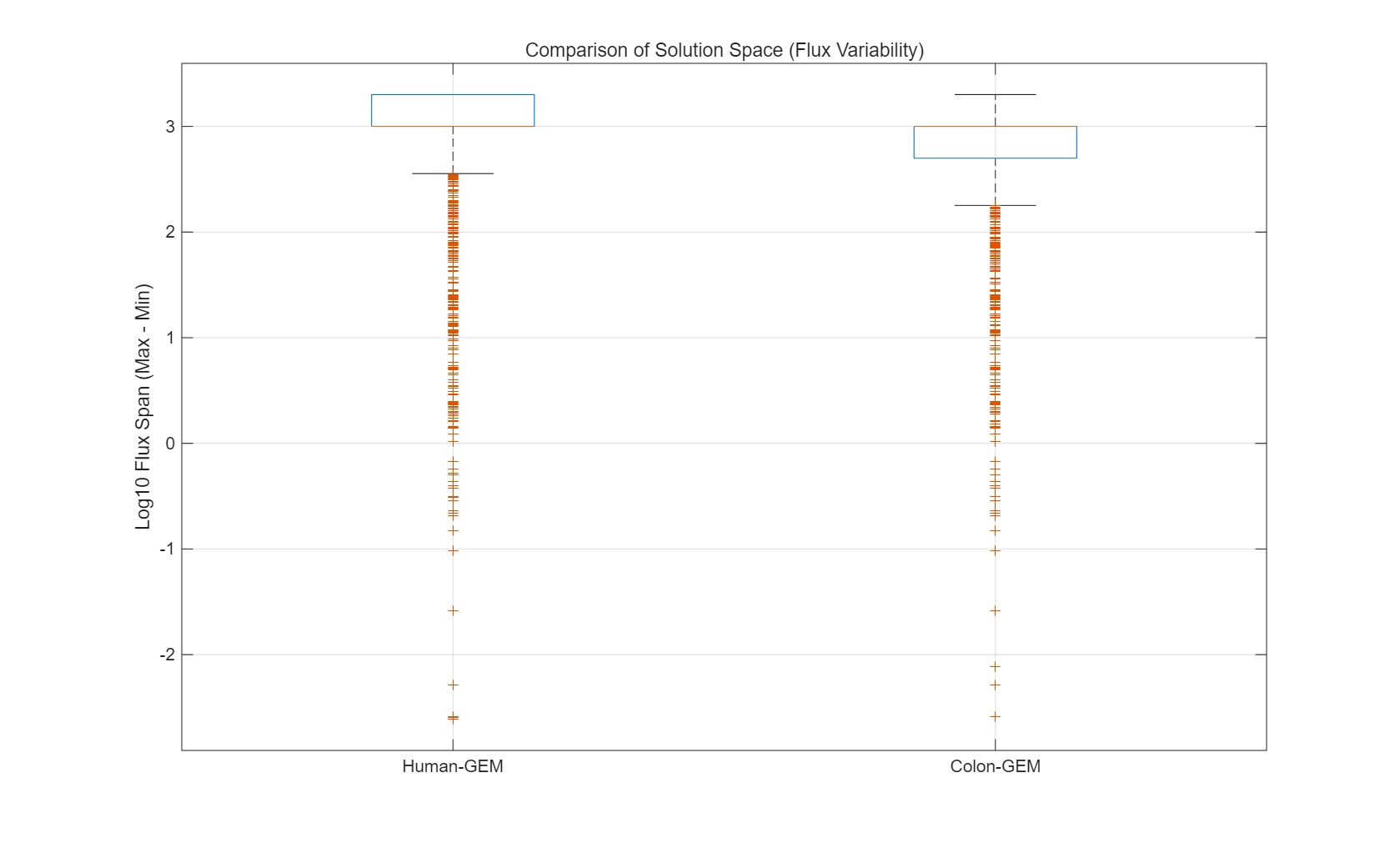

Supplement: Supplementary file 1 [file metabolites-16-00064-s001.zip › Supplementary Figure S4.jpg]
